# Metabolically healthy obesity and unhealthy normal weight rural adults in Xinjiang: prevalence and the associated factors

**DOI:** 10.1186/s12889-021-11996-y

**Published:** 2021-10-26

**Authors:** Wen-qiang Wang, Bin Wei, Yan-peng Song, Heng Guo, Xiang-hui Zhang, Xin-ping Wang, Yi-zhong Yan, Jiao-long Ma, Kui Wang, Mulatibieke Keerman, Jing-yu Zhang, Ru-lin Ma, Shu-xia Guo, Jia He

**Affiliations:** 1 Department of Public Health, Shihezi University School of Medicine, North 2th Road, Shihezi, 832000 Xinjiang China; 2 The First Affiliated Hospital of Shihezi University Medical College, Shihezi, 832000 Xinjiang China; 3 NHC Key Laboratory of Prevention and Treatment of Central Asia High Incidence Diseases (First Affiliated Hospital, School of Medicine, Shihezi University), Shihezi, 832000 Xinjiang China

**Keywords:** Metabolic status, Obesity, Normal weight, Metabolically healthy obesity, Metabolically unhealthy normal weight, Prevalence, Associated factors

## Abstract

**Background:**

This study aimed to describe the prevalence of metabolically healthy obesity (MHO) and metabolically unhealthy normal weight (MUNW) rural adults in Xinjiang and to explore their influencing factors.

**Methods:**

We selected 13,525 Uyghur, Kazakh and Han participants in Kashi, Yili and Shihezi areas in Xinjiang from 2009 to 2010. Weight status was classified according to body mass index. Metabolic phenotype was further defined based on the National Cholesterol Education Program Adult Treatment Panel III criteria.

**Results:**

The prevalence of normal weight, overweight, and obesity were 51.6, 30.2, and 14.4%, respectively. The mean age of the population was 45.04 years. The prevalence of MHO was 5.5% overall and was 38.5% among obese participants. The prevalence of MUNW was 15.5% overall and was 30.1% among normal weight participants. A metabolically healthy phenotype among obese individuals was positively associated with females and vegetable consumption ≥4 plates per week. However, this was inversely associated with higher age, red meat consumption ≥2 kg per week, and larger waist circumference (WC). Conversely, a metabolically unhealthy phenotype among normal-weight individuals was positively associated with higher age, red meat consumption ≥2 kg per week, and larger WC; this was however inversely associated with vegetable consumption ≥4 plates per week.

**Conclusions:**

The prevalence of MHO among obese adults in Xinjiang is higher than that of Han adults, while the prevalence of MUNW among normal weight adults is lower than that among Han adults. In obese and normal weight participants, higher age, more red meat consumption, and larger WC increase the risk of metabolic abnormality, and more vegetable consumption reduces the risk of metabolic abnormality.

## Background

Since the 1970s, the prevalence of obesity has risen rapidly, causing great harm to society and people’s health. This has become a major public health problem worldwide [1]. In China, from 1980 to 2015, the prevalence of obesity among adults increased significantly, that is, from 1.23 to 10.53%. In 2015, China and India had the highest numbers of obese children, whereas the United States and China had the highest numbers of obese adults [2]. Obesity is usually accompanied by a series of metabolic abnormalities, including insulin resistance (IR), triglyceride (TG) elevation, high-density lipoprotein cholesterol (HDL-C) reduction, and metabolic syndrome (MS); these are important risk factors for type 2 diabetes and cardiovascular disease [3, 4]. However, not all obese individuals show metabolic abnormalities. Some obese individuals are healthy in terms of blood pressure, blood sugar, and blood lipid levels. This type of obesity is called metabolically healthy obesity (MHO) [5]. A previous study showed that mortality due to cardiovascular disease and all-cause mortality in the MHO population was lower than that in individuals with metabolically unhealthy obesity (MUO) [6].

Conversely, some participants with normal weight have a variety of metabolic disorders [7]. These individuals are defined as the metabolically unhealthy normal weight (MUNW) phenotype [8]. Hinnouho et al. found that the risks of the cardiovascular disease and type 2 diabetes among the MUNW population were 2.04 times and 3.12 times that of the metabolically healthy normal weight (MHNW) population, respectively [9]. Previous studies show that weight control and medication can reduce adverse outcomes among the MUNW population [10, 11]. However, normal weight will always cover up their need for timely intervention, so that they face a higher risk of disease, so early identification of metabolically unhealthy normal weight population becomes particularly important [12].

Once the MHO and MUNW phenotypes were proposed, they gained a lot of attention from scholars in many countries. According to previous studies, the prevalence of MHO and MUNW ranged from 3.9 to 8.9% [13–15], and 4.3 to 17.3% respectively [16, 17]. This difference may be due to the age, gender, race, and geographic location of the participants. Additionally, many studies showed that the factors (e.g. age, sex and ethnicity) associated with MHO and MUNW were not similar [14, 15, 17, 18]. Xinjiang is located in Northwest China. The population of this area is multi-ethnic, mainly consisting of the Uyghur, Kazakh, and Han ethnic groups. The prevalence of obesity, especially the prevalence of abdominal obesity among Uyghurs (54%) and Kazakhs (60%) in rural Xinjiang were higher than those in the Chinese Han population (37.4%) [19]. However, currently, there are only a few reports on large-scale sample surveys of MHO and MUNW in Xinjiang. Therefore, this study aimed to analyze the influencing factors of MHO and MUNW among rural adults in Xinjiang, which was pertinent for the precise intervention for obesity and the early prevention of related diseases in this region.

## Materials and methods

### Study population

This study was conducted in Xinjiang from 2009 to 2010**.** A multi-stage (prefecture-county-township-village) stratified cluster random sampling method was used to select the participants. First, according to the region, ethnic distribution, and economic development, we selected three representative regions namely Kashi, Yili, and Shihezi. We then randomly selected one county in each prefecture and one township in each county. That is, we used a stratified sampling method to select the corresponding villages in each township and further investigated the residents of the selected villages. We chose individuals who met the following inclusion criteria: (1) Uyghur, Kazakh or Han residents age ≥ 18 years and living in the local area for at least 6 months (2) were free of no serious illness, and (3) willingness to cooperate in completing the investigation. Additionally, we excluded pregnant women (*n* = 135). Consequently, this study included 13,525 participants who completed the questionnaire surveys, physical examinations, and biochemical tests. The overall response rate was 89.6%. All the participants signed an informed consent form, and all the experiment protocol for involving human data was in accordance to Declaration of Helsinki.

### Anthropometric and biochemical measurement

The measurements, which included height, weight, waist circumstance (WC), systolic blood pressure (SBP), and diastolic blood pressure (DBP), was conducted by uniformly trained investigators. A height and weight scale was used to measure height and weight after the participants took their shoes, hats, and heavy clothing off. The BMI was calculated as weight (kg) divided by the square of height (m). The WC was measured using an inelastic tape at the midpoint between the iliac crest and the lowest rib. The participants sat and rested for at least 5 min after which they had their SBP and DBP measured using a mercury sphygmomanometer. Each measurement required an interval of 30 s; the average value was obtained after three measurements. After 10 h of fasting, whole blood samples were collected from each participant. Fasting plasma glucose (FPG), TG, and HDL-C were tested using an automatic biochemical analyzer (Olympus AU 2700; Olympus Diagnostics, Hamburg, Germany) at the Laboratory Department of the First Affiliated Hospital of Shihezi University School of Medicine.

### Questionnaire survey

Each participant was interviewed face-to-face. The questionnaires included general demographic data (age, gender, ethnicity, and educational level), behavior and lifestyle (smoking, drinking), and eating habits (fresh milk, eggs, fresh meat, vegetables, and fruits). Smoking was defined as current smoking with more than 100 smoked cigarettes before the time of the interview [20]. Drinking was defined as drinking alcoholic beverages (beer, red wine, and white wine) at least twice a month and continuing to drink actively [21]. A semi-quantitative food frequency method was used to investigate the dietary intake. The intake of fresh milk, meat, eggs, and fruits and vegetables was measured in liters, kilograms, numbers, and a plate with a diameter of 16 cm, respectively.

### Outcome and exposure definitions

According to the regulations of the Working Group on Obesity in China [22], the participants were defined as underweight (BMI < 18.5 kg/m^2^), normal weight (BMI 18.5–23.9 kg/m^2^), overweight (BMI 24.0–27.9 kg/m^2^), and obese (BMI ≥28.0 kg/m^2^). Abdominal obesity was defined as WC ≥85 cm and WC ≥80 cm among men and women, respectively. The MS components in the National Cholesterol Education Program Adult Treatment Panel III (2005) were used to define the metabolic status (because WC and BMI have collinearity, remove it) [23] Metabolic health and unhealth were defined as the following four metabolic abnormalities ≤1, and metabolic abnormalities > 1, respectively: (1) TG ≥1.70 mmol/L; (2) SBP ≥130 mmHg or DBP ≥85 mmHg, or have been diagnosed as hypertension; (3) FPG ≥5.6 mmol/L, or have been diagnosed as type 2 diabetes; (4) HDL-C < 1.04 mmol/L (men), HDL-C < 1.30 mmol/L (women).

### Statistical analysis

Continuous variables were expressed as mean ± standard deviation (SD), and categorical variables were expressed as numbers and percentages. The means between each group were compared using the t-test. The chi-square test and trend test were used to analyze the categorical variables. Binary logistic regression was used to analyze the factors associated with MHO and MUNW. Model 1 was adjusted for age, gender and ethnicity. Model 2 = Model 1 + educational level, smoking, drinking, fresh milk, egg, fresh meat, vegetables and fruits. The WC, which is an important indicator for measuring abdominal obesity, was also incorporated into the model 3 for adjustment. Statistical significance was defined as a two-tailed *P*-value < 0.05. All the analyses were conducted using SPSS version 20 (SPSS Inc., Chicago, IL, USA).

## Results

### Baseline characteristics of the study population

A total of 13,525 participants with an average age of 45.04 ± 14.60 years were recruited. Among the participants, 51.6% (6973) were normal weight, 30.2% (4078) were overweight, and 14.4% (1945) were obese. The prevalence of MHO was 5.5%, accounting for 38.5% of obese population. The prevalence of MUNW was 15.5%, accounting for 30.1% of the normal weight population. In each weight group, the age and WC of the metabolically unhealthy participants were higher than those of the metabolically healthy participants (*P* <  0.001). The percentage of females in the metabolically healthy group among the obese individuals was higher than that in the metabolically unhealthy group (*P* = 0.036). There were statistical differences in the percentage of education level and consumption of fresh milk, vegetables, and fruits between the healthy and unhealthy metabolic groups, for both the normal weight and overweight individuals (*P* <  0.05) (Table 1).

**Table 1:** Characteristics of the participants according to weight status and metabolic phenotype

Characteristics	Normal weight		*p* Value	Overweight		*p* Value	Obese		*p* Value
	Metabolically healthy	Metabolically unhealthy		Metabolically healthy	Metabolically unhealthy		Metabolically healthy	Metabolically unhealthy	
n (prevalence%)	4874 (36.0)	2099 (15.5)	─	2235 (16.5)	1843 (13.6)	─	749 (5.5)	1196 (8.8)	─
Age (years) mean ± SD	40.83 ± 14.75	47.45 ± 15.60	< 0.001	45.56 ± 13.03	49.27 ± 12.93	< 0.001	47.36 ± 12.46	50.97 ± 11.79	< 0.001
Gender n (%)			0.938			0.182			0.036
Male	2183 (44.8)	938 (44.7)		1030 (46.1)	888 (48.2)		289 (38.6)	519 (43.4)	
Female	2691 (55.2)	1161 (55.3)		1205 (53.9)	955 (51.8)		460 (61.4)	677 (56.6)	
Ethnicity n (%)			0.919			0.172			0.240
Kazakh	2376 (48.7)	1019 (48.5)		1054 (47.2)	880 (47.7)		447 (59.7)	757 (63.3)	
Uyghur	1488 (30.5)	636 (30.3)		440 (19.7)	322 (17.5)		100 (13.4)	137 (11.5)	
Han	1010 (20.7)	444 (21.2)		741 (33.2)	641 (34.8)		202 (27.0)	302 (25.3)	
Educational level n (%)			< 0.001			< 0.001			0.346
Illiteracy	797 (16.4)	447 (21.3)		425 (19.0)	431 (23.4)		127 (17.0)	228 (19.1)	
Primary school	1993 (40.9)	919 (43.8)		968 (43.3)	828 (44.9)		353 (47.1)	528 (44.1)	
≥ Junior high school	2084 (42.8)	733 (34.9)		842 (37.7)	584 (31.7)		269 (35.9)	440 (36.8)	
Smoking n (%)			0.305			0.599			0.812
No	3825 (78.5)	1624 (77.4)		1726 (77.2)	1436 (77.9)		584 (78.0)	938 (78.4)	
Yes	1049 (21.5)	475 (22.6)		509 (22.8)	407 (22.1)		165 (22.0)	258 (21.6)	
Drinking n (%)			0.929			0.176			0.744
No	4355 (89.4)	1877 (89.4)		1906 (85.3)	1599 (86.8)		636 (84.9)	1009 (84.4)	
Yes	519 (10.6)	222 (10.6)		329 (14.7)	244 (13.2)		113 (15.1)	187 (15.6)	
Fresh milk n (%)			0.047			0.024			0.464
no or < 0.5 L per week	3953 (81.1)	1756 (83.7)		1710 (76.5)	1476 (80.1)		558 (74.5)	899 (75.2)	
0.5 L–1.5 L per week	526 (10.8)	188 (9.0)		290 (13.0)	212 (11.5)		97 (13.0)	167 (14.0)	
≥ 1.5 L per week	395 (8.1)	155 (7.4)		235 (10.5)	155 (8.4)		94 (12.6)	130 (10.9)	
Egg n (%)			0.032			0.081			0.427
no or < 1 per week	2342 (48.1)	1091 (52.0)		1110 (49.7)	982 (53.3)		433 (57.8)	723 (60.5)	
1–3 per week	1708 (35.0)	694 (33.1)		735 (32.9)	584 (31.7)		216 (28.8)	322 (26.9)	
≥ 4 per week	824 (16.9)	314 (15.0)		390 (17.4)	277 (15.0)		100 (13.4)	151 (12.6)	
Fresh meat n (%)			0.019			0.087			< 0.001
no or < 1 kg per week	1093 (22.4)	463 (22.1)		417 (18.7)	315 (17.1)		128 (17.1)	156 (13.0)	
1-2 kg per week	1601 (32.8)	626 (29.8)		643 (28.8)	493 (26.7)		210 (28.0)	275 (23.0)	
≥ 2 kg per week	2180 (44.7)	1010 (48.1)		1175 (52.6)	1035 (56.2)		411 (54.9)	765 (64.0)	
Vegetables n (%)			< 0.001			0.002			0.080
no or < 1 plate per week	340 (7.0)	199 (9.5)		120 (5.4)	107 (5.8)		30 (4.0)	70 (5.9)	
1–3 plates per week	2358 (48.4)	1060 (50.5)		866 (38.7)	815 (44.2)		284 (37.9)	482 (40.3)	
≥ 4 plates per week	2176 (44.6)	840 (40.0)		1249 (55.9)	921 (50.0)		435 (58.1)	644 (53.8)	
Fruits n (%)			0.007			0.009			0.193
no or < 1 plate per week	978 (20.1)	492 (23.4)		456 (20.4)	442 (24.0)		169 (22.6)	315 (26.3)	
1–3 plates per week	2571 (52.7)	1055 (50.3)		1066 (47.7)	877 (47.6)		363 (48.5)	549 (45.9)	
≥ 4 plates per week	1325 (27.2)	552 (26.3)		713 (31.9)	524 (28.4)		217 (29.0)	332 (27.8)	
WC (cm) mean ± SD	79.22 ± 7.70	81.69 ± 8.37	< 0.001	87.55 ± 7.75	89.69 ± 7.93	< 0.001	97.52 ± 9.54	100.56 ± 10.26	< 0.001

*WC* waist circumstance, *SD* standard deviation

### Prevalence of MHO in obese participants

Among the 1945 obese individuals, the prevalence of MHO in males (35.8%) was lower than that in females (40.5%) (*P* = 0.036). The prevalence of MHO in males and females gradually decreased with the increase in age (*p* trend< 0.001). There was no statistical difference in the prevalence of MHO between the three ethnic groups (*P* > 0.05) (Table 2).

**Table 2:** Prevalence of metabolically healthy in obese subjects stratified by age and gender

	All subjects	Male	Female	*p* Value ^a^
Overall n (%)	749 (38.5)	289 (35.8)	460 (40.5)	0.036
Age group n (%)				
18–24	59 (62.1)	18 (48.6)	41 (70.7)	0.031
25–34	147 (47.3)	65 (43.9)	82 (50.3)	0.260
35–44	226 (40.2)	81 (36.2)	145 (42.9)	0.111
45–54	186 (33.5)	77 (32.9)	109 (34.0)	0.796
55–64	101 (32.1)	39 (30.0)	62 (33.5)	0.511
≥ 65	30 (28.0)	9 (25.7)	21 (29.2)	0.709
*p*_trend_	< 0.001	< 0.001	< 0.001	
Ethnicity n (%)				
Kazakh	447 (37.1)	164 (33.5)	283 (39.6)	0.033
Uyghur	100 (42.2)	42 (37.5)	58 (46.4)	0.166
Han	202 (40.1)	83 (40.1)	119 (40.1)	0.995
*p* Value ^b^	0.240	0.235	0.354	

^a^ Comparison of the prevalence of metabolically healthy obesity between different genders

^b^ Comparison of the prevalence of metabolically healthy obesity between different ethnicities

### Prevalence of MUNW in normal weight participants

There was no statistical difference in the prevalence of MUNW between males and females (*P* > 0.05) among the 6973 normal weight individuals. The prevalence of MUNW in males and females gradually increased (*p* trend< 0.001) with the increase in age. There was no statistical difference in the prevalence of MHO between the three ethnic groups (*P* > 0.05) (Table 3).

**Table 3:** Prevalence of metabolically unhealthy in normal weight subjects stratified by age and gender

	All subjects	Male	Female	***p*** Value ^**a**^
Overall n (%)	2099 (30.1)	938 (30.1)	1161 (30.1)	0.938
Age group n (%)				
18–24	316 (19.3)	173 (23.0)	143 (16.1)	< 0.001
25–34	367 (24.0)	164 (25.8)	203 (22.7)	0.164
35–44	440 (29.8)	171 (30.1)	269 (29.7)	0.882
45–54	466 (38.5)	177 (32.4)	289 (43.6)	< 0.001
55–64	335 (44.6)	163 (40.2)	172 (49.7)	0.009
≥ 65	175 (47.4)	90 (42.3)	85 (54.5)	0.020
*p*_trend_	< 0.001	< 0.001	< 0.001	
Ethnicity n (%)				
Kazakh	1019 (30.0)	435 (29.9)	584 (30.1)	0.915
Uyghur	636 (29.9)	334 (30.3)	302 (29.5)	0.703
Han	444 (30.5)	169 (29.9)	275 (30.9)	0.680
*p* Value ^b^	0.919	0.974	0.804	

^a^ Comparison of the prevalence of metabolically unhealthy normal weight between different genders

^b^ Comparison of the prevalence of metabolically unhealthy normal weight between different ethnicities.

### Associated factors of MHO in obese individuals

A multivariate logistic regression analysis was conducted among the obese individuals; the results showed that a metabolically healthy phenotype was positively associated with females (odds ratio [OR] = 1.42, 95% confidence interval [CI] = 1.13–1.77) and the consumption of vegetables ≥4 plates per week (OR = 1.85, 95%CI = 1.02–3.35). This was however, negatively associated with higher age and the consumption of red meat ≥2 kg per week (OR = 0.62, 95%CI = 0.45–0.85), and a larger WC. Further, the risk of MHO showed a significant downward trend as age increased. Compared with 18–24 years, the OR dropped from 0.56 in the 25–34 years group to 0.26 in the ≥65 years group (Table 4).

**Table 4:** Odds ratios for metabolically healthy phenotype associated with demographic and lifestyle factors in obese individuals

Characteristics	Unadjusted OR (95%CI)	Model 1 OR (95%CI)	Model 2 OR (95%CI)	Model 3 OR (95%CI)
Age (years)				
18–24	1.00	─	1.00	1.00
25–34	0.55 (0.34,0.88)	─	0.52 (0.32,0.85)	0.56 (0.34,0.91)
35–44	0.41 (0.26,0.64)	─	0.37 (0.23,0.59)	0.41 (0.25,0.65)
45–54	0.31 (0.20,0.48)	─	0.29 (0.18,0.46)	0.32 (0.20,0.51)
55–64	0.29 (0.18,0.46)	─	0.27 (0.17,0.45)	0.30 (0.18,0.50)
≥ 65	0.24 (0.13,0.43)	─	0.23 (0.12,0.43)	0.26 (0.14,0.49)
Gender				
Male	1.00	─	1.00	1.00
Female	1.22 (1.01,1.47)	─	1.30 (1.05,1.62)	1.42 (1.13,1.77)
Ethnicity				
Kazakh	1.00	─	1.00	1.00
Uyghur	1.24 (0.93,1.64)	─	1.06 (0.76,1.48)	1.03 (0.73,1.44)
Han	1.13 (0.92,1.40)	─	1.22 (0.93,1.61)	1.14 (0.87,1.51)
Educational Level				
Illiteracy	1.00	1.00	1.00	1.00
Primary school	1.20 (0.93,1.55)	1.24 (0.93,1.66)	1.24 (0.93,1.66)	1.26 (0.95,1.69)
≥ Junior high school	1.10 (0.84,1.43)	1.05 (0.76,1.43)	1.04 (0.75,1.43)	1.05 (0.76,1.45)
Smoking				
No	1.00	1.00	1.00	1.00
Yes	1.03 (0.82,1.28)	1.15 (0.90,1.46)	1.11 (0.85,1.43)	1.10 (0.85,1.42)
Drinking				
No	1.00	1.00	1.00	1.00
Yes	0.96 (0.74,1.24)	1.04 (0.78,1.37)	1.05 (0.78,1.40)	1.04 (0.77,1.40)
Fresh milk				
no or < 0.5 L per week	1.00	1.00	1.00	1.00
0.5 L–1.5 L per week	0.94 (0.71,1.24)	0.96 (0.72,1.28)	0.93 (0.69,1.26)	0.93 (0.69,1.26)
≥ 1.5 L per week	1.17 (0.86,1.60)	1.24 (0.90,1.69)	1.14 (0.82,1.58)	1.14 (0.82,1.58)
Egg				
no or < 1 per week	1.00	1.00	1.00	1.00
1–3 per week	1.12 (0.88,1.43)	1.06 (0.83,1.36)	1.07 (0.83,1.39)	1.05 (0.80,1.37)
≥ 4 per week	1.11 (0.80,1.52)	0.96 (0.68,1.35)	0.92 (0.64,1.33)	0.90 (0.63,1.30)
Fresh meat				
no or < 1 kg per week	1.00	1.00	1.00	1.00
1-2 kg per week	0.93 (0.68,1.28)	0.93 (0.67,1.28)	0.85 (0.61,1.20)	0.87 (0.61,1.23)
≥ 2 kg per week	0.66 (0.50,0.87)	0.66 (0.49,0.90)	0.61 (0.45,0.84)	0.62 (0.45,0.85)
Vegetables				
no or < 1 plate per week	1.00	1.00	1.00	1.00
1–3 plates per week	1.39 (0.81,2.41)	1.40 (0.79,2.49)	1.48 (0.83,2.63)	1.48 (0.83,2.64)
≥ 4 plates per week	1.59 (0.94,2.71)	1.66 (0.94,2.92)	1.84 (1.02,3.32)	1.85 (1.02,3.35)
Fruits				
no or < 1 plate per week	1.00	1.00	1.00	1.00
1–3 plates per week	1.23 (0.97,1.55)	1.16 (0.91,1.47)	1.12 (0.87,1.43)	1.11 (0.87,1.43)
≥ 4 plates per week	1.21 (0.93,1.57)	1.07 (0.82,1.40)	1.09 (0.83,1.43)	1.08 (0.82,1.43)
WC (cm)				
< 90 (men) or < 80 (women)	1.00	─	─	1.00
90–100 (men) or 80–90 (women)	0.67 (0.42,1.08)	─	─	0.71 (0.44,1.16)
≥ 100 (men) or ≥ 90 (women)	0.49 (0.31,0.76)	─	─	0.51 (0.32,0.82)

*WC* waist circumference, Model 1 was adjusted for age, gender and ethnicity, Model 2 = Model 1 + educational level, smoking, drinking, fresh milk, egg, fresh meat, vegetables and fruits, Model 3 = Model 2 + waist circumference.

### Associated factors of MUNW in normal weight individuals

A multiple-factor analysis among the normal weight individuals indicated that a metabolically unhealthy phenotype was positively associated with higher age, the consumption of red meat ≥2 kg per week (OR = 1.25, 95%CI = 1.05–1.48), and a larger WC; however, this was inversely associated with the consumption of vegetables ≥4 plates per week (OR = 0.64, 95%CI = 0.48–0.85). The risk of MUNW obviously increased with the increase in age. Compared with the 18–24 years group, the OR increased from 1.32 in the 25–34 years group to 3.33 in the ≥65 years group (Table 5).

**Table 5:** Odds ratios for metabolically unhealthy phenotype associated with demographic and lifestyle factors in normal weight individuals

Characteristics	Unadjusted OR (95%CI)	Model 1 OR (95%CI)	Model 2 OR (95%CI)	Model 3 OR (95%CI)
Age (years)				
18–24	1.00	─	1.00	1.00
25–34	1.32 (1.12,1.57)	─	1.35 (1.13,1.61)	1.32 (1.11,1.57)
35–44	1.78 (1.51,2.10)	─	1.83 (1.54,2.19)	1.74 (1.46,2.08)
45–54	2.63 (2.22,3.11)	─	2.72 (2.27,3.27)	2.50 (2.07,3.01)
55–64	3.37 (2.79,4.07)	─	3.41 (2.78,4.18)	3.10 (2.52,3.80)
≥ 65	3.78 (2.98,4.79)	─	3.70 (2.86,4.78)	3.33 (2.57,4.31)
Gender				
Male	1.00	─	1.00	1.00
Female	1.00 (0.91,1.11)	─	1.08 (0.96,1.22)	0.96 (0.85,1.09)
Ethnicity				
Kazakh	1.00	─	1.00	1.00
Uyghur	1.00 (0.89,1.12)	─	1.02 (0.88,1.19)	1.00 (0.86,1.17)
Han	1.03 (0.90,1.17)	─	0.91 (0.77,1.09)	0.97 (0.81,1.15)
Educational level				
Illiteracy	1.00	1.00	1.00	1.00
Primary school	0.82 (0.72,0.95)	0.91 (0.78,1.06)	0.91 (0.78,1.07)	0.90 (0.77,1.05)
≥ Junior high school	0.63 (0.54,0.72)	0.92 (0.78,1.09)	0.93 (0.79,1.11)	0.92 (0.78,1.10)
Smoking				
No	1.00	1.00	1.00	1.00
Yes	1.07 (0.94,1.21)	1.04 (0.91,1.20)	1.03 (0.89,1.20)	1.03 (0.89,1.19)
Drinking				
No	1.00	1.00	1.00	1.00
Yes	0.99 (0.84,1.17)	1.08 (0.90,1.29)	1.04 (0.86,1.26)	1.04 (0.86,1.26)
Fresh milk				
no or < 0.5 L per week	1.00	1.00	1.00	1.00
0.5 L–1.5 L per week	0.81 (0.63,1.02)	0.81 (0.63,1.04)	0.82 (0.64,1.06)	0.82 (0.63,1.07)
≥ 1.5 L per week	0.88 (0.71,1.10)	0.87 (0.69,1.09)	0.93 (0.72,1.20)	0.92 (0.71,1.20)
Egg				
no or < 1 per week	1.00	1.00	1.00	1.00
1–3 per week	0.87 (0.76,1.00)	0.90 (0.77,1.05)	0.90 (0.77,1.06)	0.90 (0.76,1.05)
≥ 4 per week	0.82 (0.66,1.01)	0.85 (0.68,1.08)	0.87 (0.68,1.11)	0.86 (0.67,1.10)
Fresh meat				
no or < 1 kg per week	1.00	1.00	1.00	1.00
1-2 kg per week	0.92 (0.80,1.07)	0.96 (0.82,1.12)	1.05 (0.90,1.24)	1.03 (0.88,1.22)
≥ 2 kg per week	1.09 (0.95,1.25)	1.16 (0.99,1.35)	1.27 (1.07,1.51)	1.25 (1.05,1.48)
Vegetables				
no or < 1 plate per week	1.00	1.00	1.00	1.00
1–3 plates per week	0.77 (0.61,0.96)	0.80 (0.63,1.03)	0.81 (0.62,1.06)	0.80 (0.61,1.05)
≥ 4 plates per week	0.66 (0.53,0.83)	0.66 (0.51,0.85)	0.65 (0.49,0.85)	0.64 (0.48,0.85)
Fruits				
no or < 1 plate per week	1.00	1.00	1.00	1.00
1–3 plates per week	0.82 (0.72,0.93)	0.91 (0.80,1.05)	0.96 (0.82,1.11)	0.96 (0.82,1.12)
≥ 4 plates per week	0.83 (0.72,0.96)	0.95 (0.82,1.11)	0.97 (0.82,1.13)	0.95 (0.81,1.12)
WC (cm)				
< 90 (men) or < 80 (women)	1.00	─	─	1.00
90–100 (men) or 80–90 (women)	1.46 (1.30,1.64)	─	─	1.32 (1.16,1.50)
≥ 100 (men) or ≥ 90 (women)	2.16 (1.77,2.65)	─	─	1.72 (1.38,2.14)

*WC* waist circumference, Model 1 was adjusted for age, gender and ethnicity, Model 2 = Model 1 + educational level, smoking, drinking, fresh milk, egg, fresh meat, vegetables and fruits, Model 3 = Model 2 + waist circumference.

## Discussion

Our study showed that the overall prevalence of MHO among the rural adults in Xinjiang was 5.5% and its prevalence among the obese participants was 38.5%. The factors positively associated with a metabolically healthy phenotype were younger age, female gender, smaller WC, consumption of less meat and more vegetables. The overall prevalence of MUNW among the multi-ethnic adults in Xinjiang was 15.5%, whereas its prevalence among the normal weight participants was 30.1%. The factors positively associated with a metabolically unhealthy phenotype were higher age, larger WC, consumption of more meat and less vegetables.

Mathew’s study indicated that the prevalence of MHO among men and women ranged from 3.3 to 32.1% and 12.2 to 57.5%, respectively [24]. The prevalence of MUNW in the U.S. and South Korean populations was 23.5 and 8.7%, respectively [24]. The prevalence of MHO varied significantly among different ethnicities and even in the same ethnicity, with different diagnostic criteria [25]. Approximately two-fifths of obese individuals in Xinjiang were metabolically healthy, which was higher than that of the domestic (except Xinjiang) Han ethnicity (27.9%) [15]. Furthermore, one-third of the normal weight individuals were metabolically unhealthy, which was lower than that of the domestic (except Xinjiang) Han ethnicity (34.1%) [18]. The reason behind the differences in the prevalence rates may be related to rural residents whose occupational-labor intensity is relatively high.

Many studies suggested that the metabolic status of men and women deteriorated with age, which was unrelated to weight status and the criteria for defining metabolic abnormalities [5]. This was consistent with the results of this study. One study showed that visceral fat tissue accumulates faster with age, eventually leading to metabolic abnormalities [26]. This trend in the female population may also be due to the gradual weakening of the protective effect of female estrogen on the metabolic state. Zhang et al. indicated that premenopause was an independent protective factor for MHO [27]. Our findings showed that among obese individuals, the prevalence of MHO in women was higher than that in men, which was consistent with the results of another study [17]. This may be due to gender differences in fat distribution. Male fat is supposedly more likely to accumulate around internal organs. This fat pattern was considered a high-risk body fat pattern because the hormones in this fat tissue were particularly active and secreted a variety of adipokines, which in turn led to metabolic disorders [28].

This study found that among obese people, more vegetables intake was positively correlated with MHO; among people with normal weight, more vegetables intake was negatively correlated with MUNW. This is consistent with the results of previous studies. Yoo et al. found that increased vegetable intake contributed to a reduction in the risk of MS and cardiovascular disease [29]. Visioli et al. found that the dietary fiber contained in vegetables may prevent the intestinal absorption of cholesterol and bile acids, hence improving blood lipids [30]. Another study suggested that the antioxidants contained in vegetables can improve the process of inflammation [31]. One study showed that residents in rural areas of Xinjiang consumed less vegetables than other parts of China [32]. This may be related to the fact that the transportation in this area is inconvenient and the weather is cold. This has caused difficulties in the cultivation and transportation of fresh vegetables.

Our study found that in obese individuals, more meat intake was negatively associated with MHO; in people with normal weight, more meat intake was positively associated with MUNW. Previous studies also suggested that increased meat (red and processed) intake was associated with an increased risk of MS and type 2 diabetes [33, 34]. This may be related to the large amount of heme iron in meat. One study showed that reducing the intake of heme iron will help prevent IR and type 2 diabetes [35]. Furthermore, meat contains a large amount of saturated fat. Consuming a large amount of saturated fat from meat increased plasma lipoprotein and blood pressure levels [36]. The fresh meat consumed by residents in Xinjiang is red meat, mainly beef and mutton. Our study confirmed that the proportion of residents who consumed ≥2 kg of fresh meat per week was as high as 50.6%. This may be mainly because Xinjiang is located in a livestock area and the local residents have a simple diet. Further, the meat they consume is mainly beef and mutton.

This study showed that in obese subjects, the larger WC was negatively associated with MHO; in subjects with normal weight, the larger WC was positively correlated with MUNW. This was consistent with many studies. One study from China indicated that abdominal obesity was significantly positively associated with metabolic risk factors [37]. Generally, with abdominal obesity there is higher visceral fat content. Visceral adipose tissue expressed a variety of inflammatory cytokines that damaged the insulin sensitivity [38]. Visceral adipose tissue was related to increased production of adipocytokines, changes in blood lipid levels, and reduced HDL-C [39]. Our results showed that the prevalence of abdominal obesity among rural adults in Xinjiang was significantly higher than that among Han adults in China during the same period (61.0% vs. 37.4%) [40]. It reminds the local government and health institutions to increase the frequency of health promotions and physical examinations in this area.

The main advantage of this study is that the participants were multi-ethnic adults from rural areas of Xinjiang. Additionally, epidemiological data were collected by well-trained health personnel. Furthermore, biochemical measurements were performed using standard protocols. However, this study had several limitations. First, the study lacked physical activity indicators. Second, the study lacked information on the duration of obesity, which might be tightly associated with age. Finally, this study was a cross-sectional design, and the inference of causality cannot be made. Therefore, prospective studies are needed to confirm these causal relationships.

## Conclusions

The prevalence of MHO among obese adults in Xinjiang is higher than that of Han adults, while the prevalence of MUNW among normal weight adults is lower than that among Han adults. Among obese and normal weight individuals, higher age, more red meat intake, and larger WC increase the risk of metabolic abnormalities. Furthermore, more vegetable intake reduces the risk of metabolic abnormalities. Females have lower risk of metabolic abnormalities only among obese individuals.

## Data Availability

The datasets used or analyzed during the current study are available from the corresponding author on reasonable request.

## Abbreviations

MHO: Metabolically healthy obesity
MUNW: Metabolically unhealthy normal weight
WC: Waist circumference
IR: Insulin resistance
TG: Triglyceride
HDL-C: High-density lipoprotein cholesterol
MS: Metabolic syndrome
MUO: Metabolically unhealthy obesity
MHNW: Metabolically healthy normal weight
BMI: Body mass index
SBP: Systolic blood pressure
DBP: Diastolic blood pressure
FPG: Fasting plasma glucose
SD: Standard deviation
OR: Odds ratio
CI: Confidence interval
